# Seroprevalence of neutralizing antibodies against adenovirus type 14 and 55 in healthy adults in Southern China

**DOI:** 10.1038/emi.2017.29

**Published:** 2017-06-07

**Authors:** Xuehua Zheng, Xia Rong, Ying Feng, Xikui Sun, Liang Li, Qian Wang, Min Wang, Wenkuan Liu, Chufang Li, Yiyu Yang, Rong Zhou, Jiahai Lu, Liqiang Feng, Ling Chen

**Affiliations:** 1 State Key Laboratory of Respiratory Diseases, Guangzhou Institutes of Biomedicine and Health, Chinese Academy of Sciences, Guangzhou 510530, China; 2 Department of Medical Statistics and Epidemiology, School of Public Health, Sun Yat-sen University, Guangzhou 510080, China; 3 Institute of Clinical Blood Transfusion, Guangzhou Blood Center, Guangzhou 510095, China; 4 The First Affiliated Hospital of Guangzhou Medical University, Guangzhou 510230, China; 5 Pediatric Intensive Care Unit, Guangzhou Women and Children’s Medical Center, Guangzhou 510180, China

**Keywords:** human adenovirus type 14, human adenovirus type 55, neutralizing antibody, seroprevalence, Southern China

## Abstract

Re-emerging human adenovirus types 14 (Ad14) and 55 (Ad55) have caused severe respiratory diseases and even deaths during recent outbreaks. However, the seroprevalence of neutralizing antibodies (nAbs) in healthy adults, which may reflect previous circulation and help to predict potential outbreaks, remains unclear. In this study, we established micro-neutralizing (MN) assays on the basis of recombinant Ad14 and Ad55 reporter viruses, and we investigated serum nAbs in healthy blood donors from Southern China. We found that the overall seropositive rates were 24.8% and 22.4% for Ad14 and Ad55 nAbs, respectively. The seropositive rates were low in individuals younger than 20, and they gradually increased with age. Ad55-seropositive individuals tended to have high nAb titers (>1000), while low (72–200) and moderate (201–1000) nAb levels were frequently observed in Ad14-seropositive ones. Surprisingly, the seropositive rates and nAb levels were associated with the blood type but not the gender of the blood donors, with type AB individuals displaying higher seropositive rates and nAb levels. Interestingly, a significant positive correlation was observed between Ad14 and Ad55 seroprevalence, and higher titers of nAbs were detected in double-positive individuals compared to single-positive ones. These results clarified the human humoral immune responses against Ad14 and Ad55 and revealed a low level of herd immunity in some subpopulations, which emphasized the importance of monitoring these two highly virulent adenoviruses and reinforced the development of prophylactic vaccines.

## INTRODUCTION

Human adenoviruses (AdVs) are non-enveloped, double-stranded DNA viruses belonging to *Adenoviridae*.^1^ To date, more than 69 AdV genotypes, which were classified into seven species (A to G), were identified according to their genetic, biophysical or biochemical characteristics.^2^ AdV infection causes a broad spectrum of diseases in humans, including pharyngoconjunctivitis, keratoconjunctivitis, gastroenteritis, myocarditis, acute hemorrhagic cystitis, meningoencephalitis and pneumonia, depending on the infection genotype.^3^ Although the symptoms are usually mild or self-limited in immuno-competent adults, outbreaks of acute respiratory diseases (ARDs), such as community-acquired pneumonia (CAP), have been documented in particular populations, including newborns, school students and military recruits.^2^ In immuno-comprised individuals such as patients with AIDS or individuals receiving transplants, AdV infection can lead to fatal pneumonia.^4^

Recently, two members of human AdV species B, type 14 (Ad14) and 55 (Ad55), were reported to cause severe CAP in military and civilian populations.^5, 6, 7, 8, 9^ Ad14 was first identified in an outbreak of ARDs in 1955 and then vanished for a long period.^10^ A newly emergent variant of Ad14, which was designated Ad14p1, has caused several outbreaks of acute febrile respiratory illness globally since 2006.^11, 12, 13, 14, 15^ High rates of severe illness and even deaths were reported following these outbreaks.^13, 14, 15^ Ad55, an inter-typic recombinant of Ad11 and Ad14, emerged since 2005 in Singapore and subsequently appeared in China, France and Israel.^16, 17^ As a highly virulent pathogen, Ad55 was associated with life-threatening pneumonia during recent outbreaks.^18, 19^ Their unpredictable high morbidity and mortality in otherwise healthy immuno-competent adults render Ad14 and Ad55 potential threats to public health.^20^ Although the genomic and virological characteristics of Ad14 and Ad55 have been investigated,^21, 22^ the prevalence of neutralizing antibodies (nAbs) in the general populations, which is helpful for understanding human anti-Ad14 and Ad55 immunity, remains unclear.

In this study, we established our micro-neutralizing (MN) assays on the basis of recombinant Ad14 and Ad55 reporter viruses, and we investigated the seroprevalence of nAbs against these two AdVs in healthy blood donors in Southern China. The distributions of nAbs in relation to different age, gender and blood type groups were also analyzed.

## MATERIALS AND METHODS

### Generation of recombinant Ad14 and Ad55 reporter viruses

Wild-type Ad14 (GenBank NO. JQ824845.1) and Ad55 (GenBank NO. KF911353.1) were kindly provided by Prof Qiwei Zhang (Southern Medical University, China) and Prof Chengfeng Qin (Beijing Institute of Microbiology and Epidemiology, China), respectively. In brief, wild-type Ad14 and Ad55 were propagated in A549 cells (ATCC, Manassas, VA, USA). The viral genomes were extracted and purified by sodium-dodecyl-sulfonate lysis (Sigma-Aldrich, St Louis, MO, USA) followed by phenol-chloroform extraction. The terminal regions of the Ad14 and Ad55 genomes were amplified by PCR and subcloned into pMD18T vectors (TaKaRa, Dalian, China) to obtain shuttle plasmids. The genomic plasmids pAd14 and pAd55 were then generated by homologous recombination between linearized shuttle plasmids and viral genomes in *Escherichia coli* BJ5183 competent cells (Agilent Technologies, Santa Clara, CA, USA). The fragments flanking the E3 region were subsequently amplified by PCR and subcloned into a pVAX1 plasmid (Thermo Fisher Scientific, Waltham, MA, USA) to obtain p14E3LR and p55E3LR. The E3 regions were then deleted by homologous recombination between E3LR fragments and linearized genomic plasmids to obtain pAd14ΔE3 and pAd55ΔE3. Simultaneously, the coding sequences for secreted alkaline phosphatase (SEAP) or enhanced green fluorescent protein (EGFP), which was flanked by a CMV promoter and a BGH poly(A) signal, were amplified by PCR and inserted into p14E3LR and p55E3LR to obtain shuttle reporter plasmids. Finally, pAd14-EGFP, pAd14-SEAP, pAd55-EGFP, and pAd55-SEAP were constructed by homologous recombination between linearized pAd14ΔE3 or pAd55ΔE3 and shuttle reporter plasmids. The genomes of the Ad14 and Ad55 reporter viruses were released by restricted enzyme digestion with AsiSI (New England Biolabs, Ipswich, MA, USA) and transfected into HEK293 cells (ATCC). The four reporter viruses Ad14-EGFP, Ad14-SEAP, Ad55-EGFP and Ad55-SEAP were then rescued and propagated. Purified viral stocks were obtained by cesium chloride gradient centrifugation, and the infectious virions were titrated as described.^23, 24^

### Human sample collection

Serum samples from 1009 healthy blood donors were randomly collected at the Guangzhou Blood Centre, Guangzhou, China, from August 2015 to December 2015. Any factors that could lead to a biased analysis, such as the educational background and the careers of the donors, were not considered as the criteria during the collection of blood samples. The ages of the volunteers ranged from 18 to 57 years old. The percentages of males to females were 31.7% and 68.3%. The percentages of blood types A, B, AB and O were 24.4%, 41.2%, 11.5% and 22.9%, respectively. The research using human samples was approved by the Ethics Committee of Guangzhou Institutes of Biomedicine and Health (GIBH). Informed consent was obtained from each volunteer.

### Micro-neutralizing (MN) assays

The titers of nAbs that acted against Ad14 and Ad55 were detected using MN assays as previously described.^23, 24^ In brief, the HEK293 cells were seeded into 96-well plates at 3 × 10^4^ cells per well. One day later, serial dilutions of the human serums were heat-treated at 56 °C for 90 min and incubated with Ad14-SEAP or Ad55-SEAP at 4 × 10^6^ viral particles per well for 1 h at 37 °C. The mixtures were subsequently added to the 96-well plates and incubated for 24 h at 37 °C. Finally, the supernatants were harvested, and the SEAP activity was detected using the Phospha-Light System according to the manufacturer’s instructions (Thermo Fisher Scientific). The relative light units (RLUs) were recorded and the titers were calculated as the dilutions that inhibited 50% RLU values. The values <72, 72–200, 201–1000 and >1000 were denoted as negative, low, moderate and high nAb titers, respectively. To validate the feasibility of SEAP activity-based MN assays, HEK293 cells were seeded into 96-well plates at 3 × 10^4^ cells per well. Twenty-four hours later, serial dilutions of serum samples with low, moderate or high titers of Ad14 or Ad55 nAbs were incubated with Ad14-EGFP or Ad55-EGFP (4 × 10^6^ viral particles per well), respectively. Subsequently, the mixtures were added to the plates. At 24 h after infection, EGFP-expressing cells were imaged using fluorescence microscopy.

### Statistical analysis and figure development

Seroprevalence comparisons for different groups were conducted using a χ^2^-test. The trends in the seropositive rates throughout the groups were analyzed by using the Trend *χ*^2^-test. Comparisons of nAb titers among groups were performed by Mann–Whitney test. The trends in the nAb titers throughout the groups were analyzed by Kruskal–Wallis Test. All the statistical analyses were computed with SPSS version 13.0 (SPSS Inc., Chicago, IL, USA), and *P* values of less than 0.05 were considered statistically significant. The graphs were generated with GraphPad Prism version 5 (GraphPad Software, La Jolla, CA, USA) and Photoshop version 8 (Adobe Systems Incorporated, San Jose, CA, USA).

## RESULTS

### Establishment and validation of MN assays based on recombinant Ad14 and Ad55 reporter viruses

**Figure 1:**
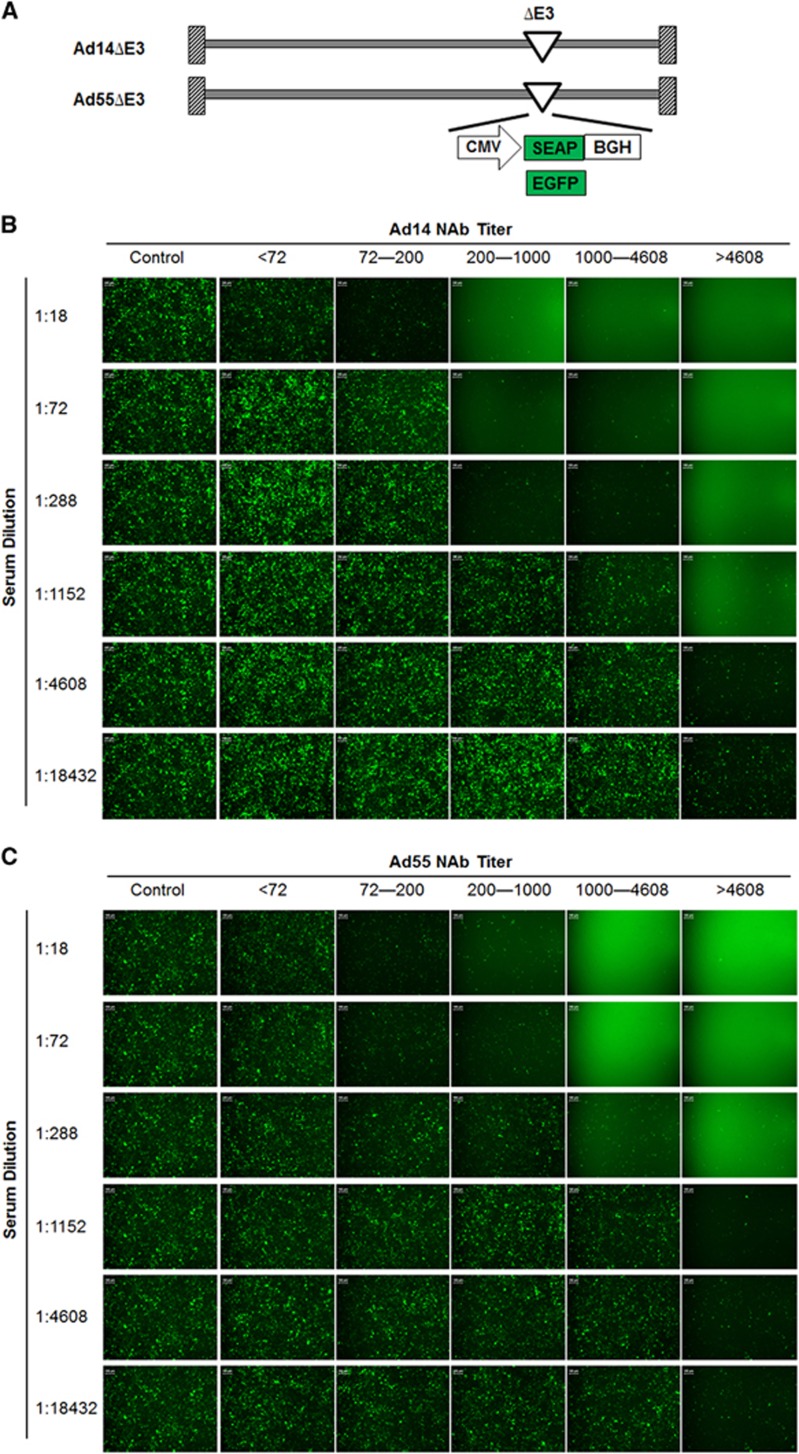
The validation of micro-neutralizing assays based on Ad14 and Ad55 reporter viruses. (**A**) A diagram of the genomic structures for Ad14 and Ad55 reporter viruses. (**B** and **C**) Serial dilutions of Ad14 (**B**) or Ad55 (**C**) -seropositive serums with nAb titers <72, 72–200, 201–1000, 1001–4608 and >4608 were incubated with Ad14-EGFP or Ad55-EGFP, and then infected into HEK293 cells. EGFP-expressing cells were imaged by fluorescence microscopy at 24 h post infection. Serums from mice that were immunized with empty Ad5 vectors were used as negative controls. This figure is representative of two independent experiments.

To generate replication-competent reporter viruses, the E3 regions of Ad14 and Ad55 were depleted, and the expression cassettes for EGFP- or SEAP were inserted (Figure 1A). The four reporter viruses Ad14-EGFP, Ad14-SEAP, Ad55-EGFP and Ad55-SEAP, were successfully obtained. The reporter genes were efficiently expressed in cells that were infected by these viruses (data not shown).

To validate the feasibility of the MN assays based on Ad14-SEAP and Ad55-SEAP, serums with different nAb titers (<72, 72–200, 201–1000, 1001–4608 and >4608) were serially diluted, incubated with Ad14-EGFP or Ad55-EGFP and infected into HEK293 cells. The results showed that serums with nAb titers <72 displayed no detectable inhibition in relation to Ad14-EGFP or Ad55-EGFP infection for all the dilutions, while those with nAb titers >72 exhibited various neutralizing activities, which was consistent with the titers that were determined by SEAP activity (Figures 1B and 1C). In addition, the MN assays based on SEAP or EGFP reporter genes were much more sensitive than the ones based on cytopathic effects (CPEs) (Supplementary Figure S1). These results suggested that the MN assays based on Ad14-SEAP and Ad55-SEAP were feasible.

### The overall seroprevalence of Ad14 and Ad55 nAbs in healthy blood donors from Southern China

**Figure 2:**
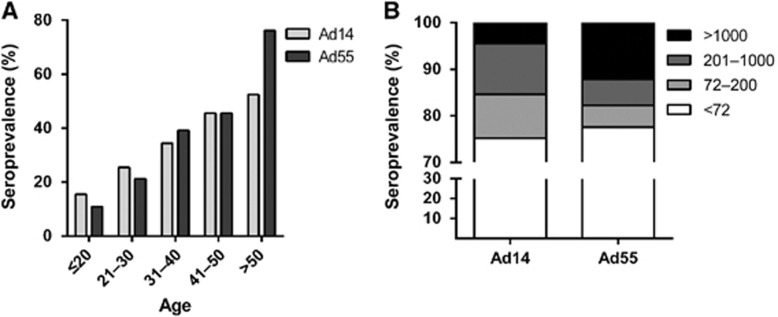
Seroprevalence of Ad14 and Ad55 nAbs for healthy adults in Southern China. Serum samples from 1009 healthy blood donors were detected using MN assays based on Ad14-SEAP and Ad55-SEAP viruses. (**A**) The overall seropositive rates of Ad14 and Ad55 nAbs by age groups. The data were analyzed by χ^2^-test. (**B**) The distributions of serums with different nAb titers.

By using Ad14-SEAP and Ad55-SEAP, the nAbs in the serums from 1009 healthy blood donors were detected (Supplementary Tables S1 and S2). The overall seroprevalence levels of Ad14 and Ad55 nAbs were 24.8% (95% confidence interval, CI: 22.1%–27.5%) and 22.4% (95% CI: 19.8%–25.0%), respectively (Supplementary Tables S1 and S2). The trends in the Ad14 and Ad55-seropositive rates were similar in this cohort, and both increased with age (Trend Chi-square test, Ad14: *P*-value <0.001; Ad55: *P*-value <0.001; Figure 2A). Notably, the seropositive rates in individuals below 20 years of age were extremely low for both Ad14 and Ad55 (15.5% and 10.9%, respectively; Figure 2A), revealing that the infections by these two AdVs and the circulation of nAbs in young adults were relatively rare. The donors were categorized into four groups according to their nAb titers as follows: negative, <72; low, 72–200; moderate, 200–1000; and high, >1000. The distributions of the different nAb titers in the whole cohort were analyzed. Moderate and low nAb titers were frequently detected in Ad14-seropositive donors, while the majority of Ad55-seropositive ones had high nAb titers (Figure 2B), suggesting that Ad55 infection might elicit stronger nAb responses in comparison with Ad14 infection.

### Titer distributions of Ad14 and Ad55 nAbs in different age groups

**Figure 3:**
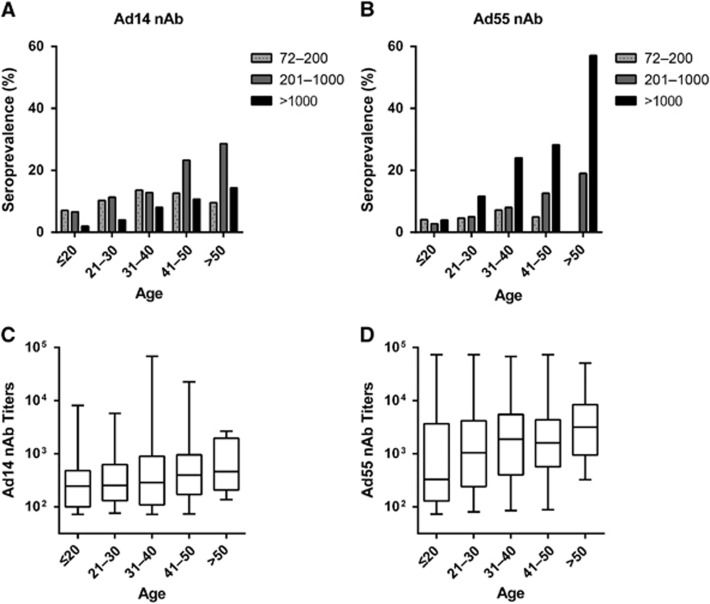
Titer distributions of Ad14 and Ad55 nAbs in age groups. The Ad14 (**A** and **C**) and Ad55 (**B** and **D**) seropositive donors were categorized into three subgroups according to their nAb titers as follows: low, 72–200; moderate, 201–1000; and high, >1000. The percentages of these subgroups in different age groups are shown (**A** and **B**), and the trends were analyzed with Trend χ^2^-test. The overall nAb levels in different age groups are also shown (**C** and **D**) and were analyzed by Kruskal–Wallis Test.

We then analyzed the distributions of nAb titers by age group. The frequency of serum samples with moderate or high nAb titers increased with age (Trend χ^2^-test, *P*-value <0.001 for moderate and high Ad14 and Ad55 nAbs, respectively), while the frequency for low nAb titers increased before 40 years old and decreased thereafter for both Ad14 and Ad55 (Figures 3A and 3B). Individuals with moderate nAb levels were dominant in Ad14-seropositive individuals older than 40 (Figure 3A), whereas the majority of Ad55-seropositive individuals older than 20 had high nAb titers (Figure 3B). Interestingly, almost 60% of volunteers older than 50 generated high levels of Ad55 nAb, and none of the individuals in this subgroup had a low titer of Ad55 nAb (Figure 3B). Thus, the overall seroprevalence levels for Ad14 and Ad55 nAbs were comparable, but their titer distributions within different age groups were dramatically different. Although no significant difference was observed for the Ad14 nAb levels in different age groups (Kruskal–Wallis test, *P*-value=0.07; Figure 3C), the Ad55 nAb levels definitely increased with age (Kruskal–Wallis test, *P*-value <0.05; Figure 3D), suggesting that nAb responses to Ad55 but not Ad14 infection tended to be enhanced when these individuals were re-infected by these strains.

### Seropositive rate and titer distributions of Ad14 and Ad55 nAbs in different gender and blood type groups

**Figure 4:**
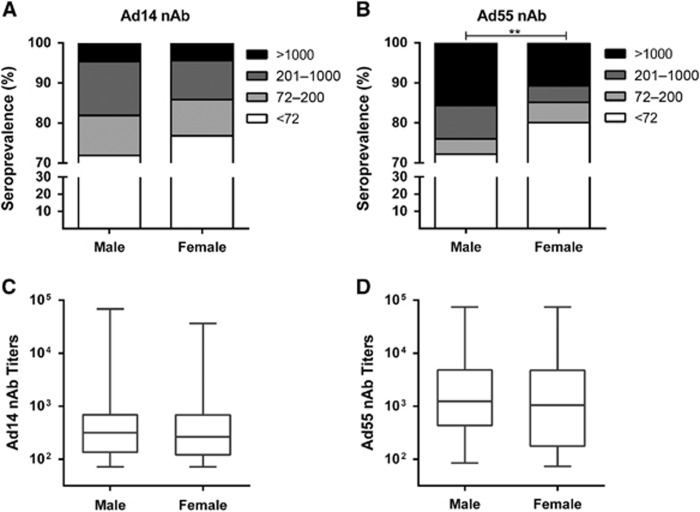
Seroprevalence and titer distributions of Ad14 and Ad55 nAbs in gender groups. The seropositive rates of Ad14 (**A**) and Ad55 (**B**) nAbs in different gender groups are shown. The difference in the overall seropositive rates between males and females was analyzed with χ^2^-test, and a *P*-value <0.05 was considered statistically significant. **P-value<0.01. The overall Ad14 (**C**) and Ad55 (**D**) nAb levels in the gender groups are shown, and the difference between males and females was analyzed by Mann–Whitney test.

**Figure 5:**
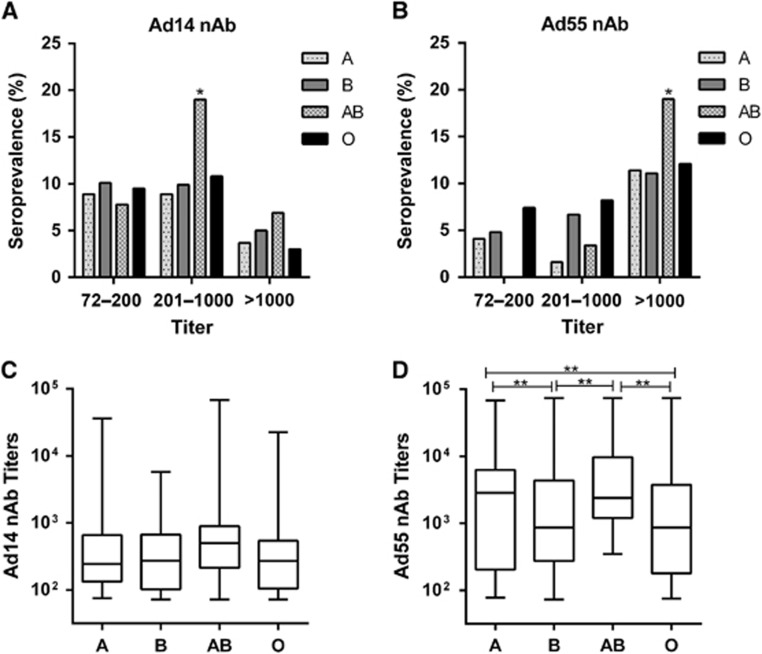
Seroprevalence and titer distributions of Ad14 and Ad55 nAbs in the blood type groups. The frequency of Ad14 (**A**) and Ad55 (**B**)-seropositive samples with different nAb titers in the blood type groups are shown. The difference in the frequencies between blood type groups was analyzed by χ^2^-test. A *P*-value <0.05 was considered to be statistically significant, *P-value <0.05. The overall Ad14 (**C**) and Ad55 (**D**) nAb levels in the blood type groups are shown, and the difference between the groups was analyzed by Mann–Whitney test. A *P*-value <0.05 was considered statistically significant, **P-value<0.01.

There was no significant difference for the Ad14-seropositive rates in the gender groups (χ^2^-test, *P*-value=0.093; Figure 4A), whereas a higher Ad55-seropositive rate was detected in males compared to females (χ^2^-test, *P*-value <0.01; Figure 4B). The titers of either Ad14 or Ad55 nAb were comparable in males and females (Mann–Whitney Test, *P*-value=0.552 and 0.600 for Ad14 and Ad55 nAb, respectively; Figures 4C and 4D), suggesting that the gender was not a major impacting factor on either the Ad14 or Ad55 nAb levels. Notably, the frequency of Ad14-seropositive samples, especially those with moderate nAb titers, was significantly higher in the AB blood type group than in the other blood type groups (χ^2^-test, *P*-value <0.05; Figure 5A). In addition, the percentage of Ad55-seropositive samples with high nAb titers was also higher in this group (χ^2^-test, *P*-value <0.05; Figure 5B). In addition, none of the volunteers with blood type AB had low Ad55 nAbs, and significantly fewer blood type A or AB donors generated moderate Ad55 nAbs (Figure 5B), which might imply that once the individuals with blood type AB were infected by Ad55, high titers of nAbs would be elicited. Consistent with this point, although the AB blood type individuals only had slightly higher levels of Ad14 nAb compared to other blood type groups (Kruskal–Wallis test, *P*-value=0.086; Figure 5C), the titer of Ad55 nAb in this group was significantly higher than that of the blood type B or O groups (Mann–Whitney test, *P*-value <0.05; Figure 5D). These results suggested that the blood type may influence Ad14 and Ad55 infection and the generation of nAb responses to these two AdVs.

### Seroprevalence of Ad14 nAb in Ad55-seropositive donors was higher than that in Ad55-seronegative ones, and vice versa

**Figure 6:**
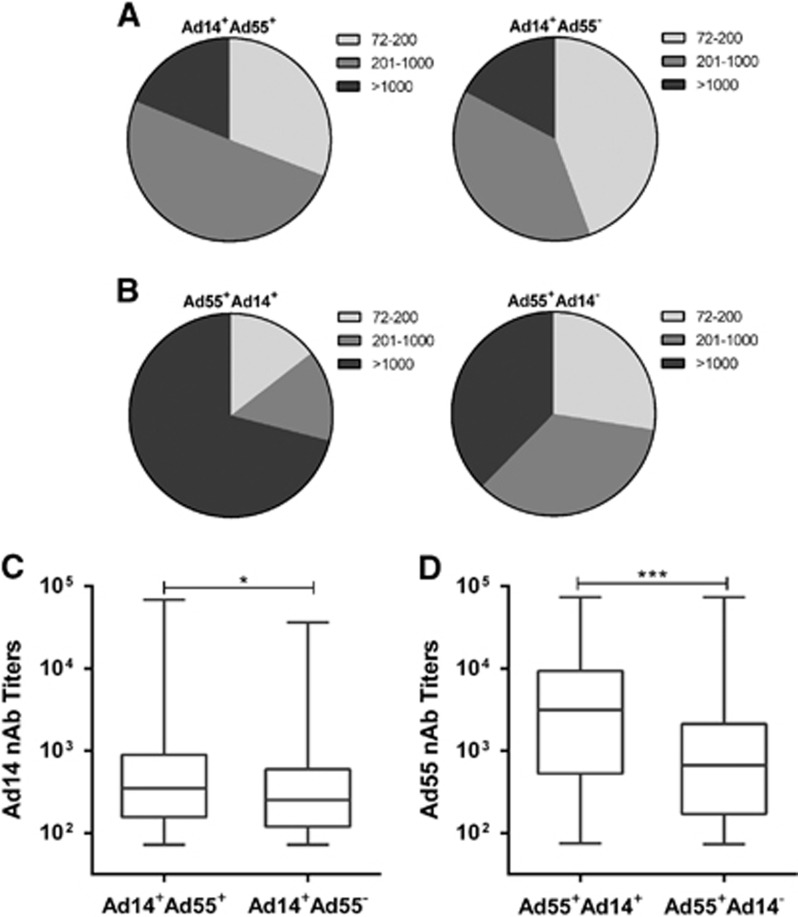
Profiling of the seropositive rates and nAb levels in single- and double-positive blood donors. (**A**) The distributions of Ad14-seropositive donors with different nAb titers in Ad55-seropositive or seronegative groups. (**B**) The distributions of Ad55-seropositive donors with different nAb titers in Ad14-seropositive or seronegative groups. The difference between the groups was analyzed by χ^2^-test. The overall Ad14 (**C**) and Ad55 (**D**) nAb levels in single- or double-positive groups are also shown. The comparison between groups was performed by Mann–Whitney test, and a *P*-value <0.05 was considered statistically significant. *P<0.05; ***P<0.001.

Next, we analyzed the frequency of Ad14-seropositive donors in Ad55-seropositive or seronegative individuals. The Ad14-seropositive rate was significantly higher in Ad55-seropositive donors than in Ad55-seronegative ones (Mann–Whitney test, *P*-value <0.01; Table 1). Similar trends were detected for the Ad55-seropositive rate in Ad14-seropositive or seronegative donors (*P*-value <0.01; Table 1). The frequency of blood donors with moderate Ad14 nAb levels was slightly higher in Ad55-seropositive compared to Ad55-seronegative donors (Figure 6A). However, in Ad14-seropositive donors, many more individuals had high titers of Ad55 nAb in comparison with Ad14-seronegative ones (χ^2^-test, *P*-value <0.001; Figure 6B). Interestingly, the titers of Ad14 nAb in Ad55-seropositive donors were significantly higher than they were in Ad55-seronegative ones (Mann–Whitney test, *P*-value <0.05; Figure 6C). In addition, the Ad55 nAb titers in Ad14-seropositive donors were much higher than they were in Ad14-seronegative donors (*P*-value <0.001; Figure 6D). All these results suggested that Ad55-seropositive donors tended to be Ad14-seropositive and *vice versa*, and double-positive serums had higher nAb titers than single-positive ones.

## DISCUSSION

Recently, two highly virulent AdVs called Ad14 and Ad55 have attracted increasing attention; these viruses caused severe clinical consequences in immuno-competent adults over a series of outbreaks.^2^ Clarifying the circulation of Ad14 and Ad55 nAbs in general populations is essential for understanding the herd immunity. Here, we investigated the seroprevalence of Ad14 and Ad55 nAbs in a large cohort from Southern China, and we found that the seropositive rates of Ad14 and Ad55 were much lower than that of Ad5 according to our previous results.^24^ This finding may imply that the general populations, especially those younger than 40, were threatened by these two AdVs. Therefore, Ad14 and Ad55 should be taken into account when patients with severe ARDs are hospitalized.^2^

In this study, we adopted EGFP- or SEAP-expressing reporter viruses based on clinical isolates of Ad14 and Ad55, and we established sensitive and high-throughput MN assays for assessing the nAbs against these two AdVs (Figure 1). These assays possessed at least two advantages over the methods that were described previously.^25^ Because the nAbs against AdV could be raised against hexon as well as fiber proteins, the chimeric Ad3 that carried the Ad14 hexon as well as Ad3 fiber proteins could only detect Ad14 nAbs that were specific for hexon.^26^ In addition, MN assays based on SEAP-expressing AdVs were reported to be more sensitive and objective than previously used CPE-based methods.^27^ Consistent results were obtained when SEAP and EGFP reporter viruses were applied (Figure 1), reflecting the feasibility of these MN assays. Given that nAbs have shown beneficial effects for treating patients infected with H5N1 influenza virus or Ebola virus,^28, 29^ these MN assays could be utilized to screen serums containing high levels of nAbs or monoclonal nAbs against Ad14 and Ad55, to treat patients with severe illnesses caused by these two AdVs (unpublished data).

For the purpose of genetic vector development, numerous studies have investigated the pre-existing nAbs against human and chimpanzee AdVs.^24, 26, 27, 30, 31, 32, 33, 34^ Regarding Ad14 and Ad55, although several case reports or epidemiological studies have been described,^5, 6, 7, 8, 9, 11, 12, 13, 14, 35^ the surveillance of their pre-existing nAbs was seldom performed. One recent study described seropositive rates of Ad55 nAbs in pediatric patients, and another study analyzed the Ad14 nAbs in a small population.^25, 36^ However, the seroprevalence of Ad14 and Ad55 nAbs in healthy adults remained to be explored, and it relied on randomly collected large-scale cohorts. Moreover, detailed analyses within age, gender or blood type groups could not be performed due to the limited samples in these two studies. Here, we investigated the seroprevalance of Ad14 and Ad55 nAbs based on a randomly collected cohort including up to 1009 healthy blood donors, and we found that the seropositive rates in young adults were low and increased with age (Figure 2), which was in accordance with the findings observed in other AdVs.^32^ The seropositive rates and nAb titers in different subgroups were successfully analyzed. We also found that Ad14-seropositive donors tended to generate moderate nAb titers while Ad55-seropositive ones were prone to having high nAb titers, especially in individuals older than 20 (Figure 3). These findings added to our knowledge of human immunity against Ad14 and Ad55 infections.

Similar to the results of other studies,^33, 37^ the seroprevalence of Ad14 and Ad55 nAbs was independent of the donor’s gender (Figure 4). However, our results showed that the blood type significantly impacted the susceptibility and nAb responses of individuals who were exposed to Ad14 or Ad55 infection (Figure 5). Although a relationship between the blood type and AdV infection was not described previously, the histo-blood group antigens were involved in the infection of hepatitis B virus and norovirus.^38, 39, 40^ We showed that individuals with blood type AB displayed the highest seropositive rates for Ad14 and Ad55 nAbs (Figure 5), indicating that this group of people might be more susceptible to infection by Ad14 or Ad55. In addition, the nAb titers in AB blood type donors were higher than that of other groups, revealing that stronger immune responses were induced by Ad14 or Ad55 infection in these individuals.

We also showed that Ad14-seropositive donors tended to have higher Ad55-seropositive rates and nAb titers than Ad14-seronegative ones, and vice versa (Figure 6), indicating that there was a positive correlation between Ad14 and Ad55 seroprevalence. The nAb titers in double-positive donors were significantly higher than they were in Ad14 or Ad55 single-positive ones (Figure 6). These results may be attributed to fiber protein, which is one of the major capsid antigens of AdVs. As a recombinant of Ad11 and Ad14, Ad55 shares a similar hexon protein with Ad11 and a fiber protein with Ad14.^21^ Although many studies reported that hexon-specific antibodies were predominantly elicited in individuals who were infected by other AdVs,^41, 42^ fiber-specific nAbs were also frequently detected.^26, 43, 44^ If fiber-specific nAbs were generated in individuals who were infected by either Ad14 or Ad55, it may neutralize both variants and thus contribute to the cross-neutralizing activity of double-positive serums. However, these speculations should be confirmed in future studies.

In summary, we established convenient MN assays to survey the effects of nAbs against Ad14 and Ad55, and we investigated the seroprevalence of these two AdVs in a general population from Southern China. We also provided the distributions of nAbs by age, gender and blood type groups. These results emphasized the importance of monitoring these two AdVs in general populations and developing vaccines for disease prevention.

## Figures and Tables

**Table 1:** Correlation of Ad14 and Ad55 nAb seropositive rates

	**Ad14-seropositive**^a^	**Ad14-seronegative**^b^	**Ad14 positive rate (%)**^c^
Ad55-seropositive^a^	117	109	51.8^d^
Ad55-seronegative^b^	133	650	17.0
Ad55 positive rate (%)^e^	46.8^d^	14.4	

a The absolute number of serums containing Ad14 or Ad55 nAbs.

b The absolute number of serums without Ad14 or Ad55 nAbs.

c The rates of Ad14-seropositive serums in Ad55-seropositive or negative ones.

d Statistics were performed by χ^2^-test, *P*<0.01.

e The rates of Ad55-seropositive serums in Ad14-seropositive or negative ones.
