# Supplementary material for: Seroprevalence of neutralizing antibodies against adenovirus type 14 and 55 in healthy adults in Southern China
Source: Emerg Microbes Infect. 2017 Jun 7;6(6):e43–. doi: 10.1038/emi.2017.29 (PMC5520307; doi:10.1038/emi.2017.29)
Supplement: Supplementary Figure S1 [file emi201729x1.pdf]

Supplementary Figure S1

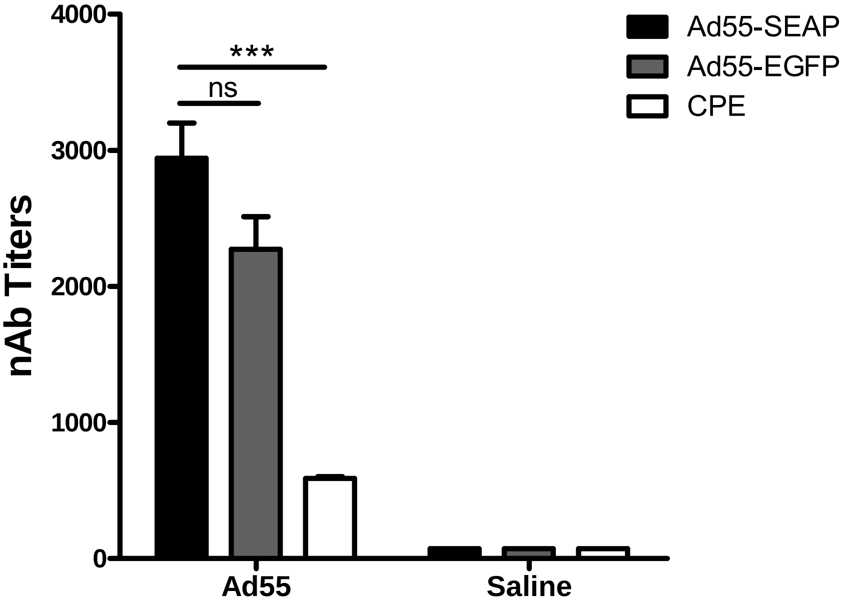

Figure S1. MN assay based on Ad55-SEAP or Ad55-EGFP was more sensitive than that based on CPE. Serums of mice immunized with inactivated Ad55 viral particles or saline were serially diluted and incubated with Ad55-SEAP, Ad55-EGFP, or a replication-competent Ad55ΔE3. The mixtures were infected into HEK293 cells. 24 hours later, SEAP activity in the mediums of Ad55-SEAP-infected cells and EGFP in the Ad55- EGFP-infected cells were detected. 7 days later, the CPE phenomenon in Ad55ΔE3-infected cells were recorded. The nAb titers were calculated as the dilutions that inhibited 50% of reporter gene expression or CPE. The comparison between groups was performed with Mann-Whitney test, and a *P*-value < 0.05 was considered statistically significant. ***, P < 0.001. ns, not significant.
