# Supplementary material for: Seroprevalence of neutralizing antibodies against adenovirus type 14 and 55 in healthy adults in Southern China
Source: Emerg Microbes Infect. 2017 Jun 7;6(6):e43–. doi: 10.1038/emi.2017.29 (PMC5520307; doi:10.1038/emi.2017.29)
Supplement: Supplementary Table S1 [file emi201729x2.docx]

**Supplementary Table S1**

**Ad14 nAb seroprevalence in the healthy adults from Guangzhou, Southern China.**

|  | **Ad14 neutralizing antibody titer. Num (%)*^a^*** | | | | **Total (Positive%)** |
| --- | --- | --- | --- | --- | --- |
|  | **<72** | **72-200** | **201-1000** | **>1000** | **≥72** |
| **Age*^b^*** |  |  |  |  |  |
| ≤20 | 387(84.5) | 32(7.0) | 30(6.6) | 9(2.0) | 71(15.5) |
| 21-30 | 225(74.5) | 31(10.3) | 34(11.3) | 12(4.0) | 77(25.5) |
| 31-40 | 82(65.6) | 17(13.6) | 16(12.8) | 10(8.0) | 43(34.4) |
| 41-50 | 55(53.4) | 13(12.6) | 24(23.3) | 11(10.7) | 48(45.6) |
| ≥50 | 10(47.6) | 2(9.5) | 6(28.6) | 3(14.3) | 11(52.4) |
| **Total** | **759(75.2)** | **95(9.4)** | **110(10.9)** | **45(4.5)** | **250(24.8)** |
|  |  |  |  |  |  |
| **Gender** |  |  |  |  |  |
| Male | 230(71.9) | 32(10.0) | 43(13.4) | 15(4.7) | 90(28.1) |
| Female | 529(76.8) | 63(9.1) | 67(9.7) | 30(4.4) | 160(23.2) |
| **Total** | **759(75.2)** | **95(9.4)** | **110(10.9)** | **45(4.5)** | **250(24.8)** |
|  |  |  |  |  |  |
| **Blood type** |  |  |  |  |  |
| A | 193(78.5) | 22(8.9) | 22(8.9) | 9(3.7) | 53(21.5) |
| B | 312(75.0) | 42(10.1) | 41(9.9) | 21(5.0) | 104(25.0) |
| AB | 77(66.4) | 9(7.8) | 22(19.0) | 8(6.9) | 39(33.6) |
| O | 177(76.6) | 22(9.5) | 25(10.8) | 7(3.0) | 54(23.4) |
| **Total** | **759(75.2)** | **95(9.4)** | **110(10.9)** | **45(4.5)** | **250(24.8)** |

*^a^* The absolute number and the percentage in the respective subgroups were shown.

*^b^* The age of serum donors ranged from 18 to 57 years old.
