# Supplementary material for: Seroprevalence of neutralizing antibodies against adenovirus type 14 and 55 in healthy adults in Southern China
Source: Emerg Microbes Infect. 2017 Jun 7;6(6):e43–. doi: 10.1038/emi.2017.29 (PMC5520307; doi:10.1038/emi.2017.29)
Supplement: Supplementary Table S2 [file emi201729x3.docx]

**Supplementary Table S2**

**Ad55 nAb seroprevalence in the healthy adults from Guangzhou, Southern China.**

|  | **Ad55 neutralizing antibody titer. Num (%)*^a^*** | | | | **Total (Positive%)** |
| --- | --- | --- | --- | --- | --- |
|  | **<72** | **72-200** | **201-1000** | **>1000** | **≥72** |
| **Age*^b^*** |  |  |  |  |  |
| ≤20 | 408(89.1) | 19(4.1) | 13(2.8) | 18(3.9) | 50(10.9) |
| 21-30 | 238(78.8) | 14(4.6) | 15(5.0) | 35(11.6) | 64(21.2) |
| 31-40 | 76(60.8) | 9(7.2) | 10(8.0) | 30(24.0) | 49(39.2) |
| 41-50 | 56(54.4) | 5(4.9) | 13(12.6) | 29(28.2) | 47(45.6) |
| ≥50 | 5(23.8) | 0(0) | 4(19.0) | 12(57.1) | 16(76.2) |
| **Total** | **783(77.6)** | **47(4.7)** | **55(5.5)** | **124(12.3)** | **226(22.4)** |
|  |  |  |  |  |  |
| **Gender** |  |  |  |  |  |
| Male | 231(72.2) | 12(3.8) | 27(8.4) | 50(15.6) | 89(27.8) |
| Female | 552(80.1) | 35(5.1) | 28(4.1) | 74(10.7) | 137(19.9) |
| **Total** | **783(77.6)** | **47(4.7)** | **55(5.5)** | **124(12.3)** | **226(22.4)** |
|  |  |  |  |  |  |
| **Blood type** |  |  |  |  |  |
| A | 204(82.9) | 10(4.1) | 4(1.6) | 28(11.4) | 42(17.1) |
| B | 322(77.4) | 20(4.8) | 28(6.7) | 46(11.1) | 94(22.6) |
| AB | 90(77.6) | 0(0) | 4(3.4) | 22(19.0) | 26(22.4) |
| O | 167(72.3) | 17(7.4) | 19(8.2) | 28(12.1) | 64(27.7) |
| **Total** | **783(77.6)** | **47(4.7)** | **55(5.5)** | **124(12.3)** | **226(22.4)** |

*^a^* The absolute number and the percentage in the respective subgroups were shown.

*^b^* The age of serum donors ranged from 18 to 57 years old.
